# Use of a natural rock material as a precursor to inhibit corrosion of Ti alloy in an aggressive phosphoric acid medium

**DOI:** 10.1038/s41598-024-60403-0

**Published:** 2024-04-29

**Authors:** Amany M. Fekry, Inna V. Filippova, Shymaa S. Medany, Soha A. Abdel-Gawad, Lev O. Filippov

**Affiliations:** 1 Université de Lorraine, CNRS, GeoRessources, 54000 Nancy, France; 2 Chemistry Department, Faculty of Science, Cairo University, Giza, 12613 Egypt; 3 Faculty of Postgraduate Studies for Nanotechnology, Cairo University, Giza, Egypt

**Keywords:** Magnesite, Corrosion inhibitor, Passive film, Ti alloy, EIS measurements, H_3_PO_4_ treatment, Chemistry, Materials science

## Abstract

The mechanism of interaction between magnesite mineral and phosphoric acid (0.001–0.5 M) in addition to the determination of the protective properties for Ti alloy (working electrode) in phosphoric acid both with and without an inhibitor have been investigated by electrochemical impedance spectroscopy (EIS) and potentiodynamic polarization measurements. Results of electrochemical tests show that the corrosion resistance of titanium alloy in phosphoric acid solution only increased and hydrogen production decreased by either decreasing acid concentration or increasing immersion time associated with the thickening of the oxide film formed on the alloy surface. On adding magnesite, the corrosion resistance of Ti alloy is enhanced by increasing the phosphoric acid concentration (0.001–0.5 M) due to the formation of sparingly soluble magnesium phosphate film on the alloy surface that inhibits the effect of increasing hydrogen evolution reaction due to the pH value decreases. The increasing adsorption behavior of the magnesite inhibitor and decreasing its diffusion were deduced from EIS measurements. Thus, the addition of 3% magnesite minimizes the corrosion by forming a new protective film (Mg_3_(PO_4_)_2_), which differs from the traditional passive film and prevents the effect of the increase of hydrogen evolution. The surface morphology and chemical composition of the tested alloy were determined using scanning electron microscopy (SEM), Fourier transform Infra-Red spectroscopy (FTIR), X-ray diffraction (XRD), X-ray Fluorescence (XRF) and In situ Raman spectroscopy.

## Introduction

Titanium and titanium alloys are widely used as construction materials in a variety of industrial applications. The high specific strength, excellent mechanical properties, and great corrosion resistance make them ideal for aerospace, transportation, power generating, and chemical industries^1^. Titanium alloys have a high strength-to-weight ratio, making them ideal for automobile and motorbike racing. The food industry, oil refineries, and desalination all use titanium^2–5^. In medical applications, metallic materials are increasingly being used as dental and orthopedic implants to restore lost functionality or to replace organs that aren't working properly. Due to its biological benefits and tolerable degradation rate, titanium alloys are one of the most often used metallic biomaterials, notably in orthopedics^6^. Titanium, on the other hand, is corroded by hot, concentrated, low-pH chloride salts. Warm or intense hydrochloric, phosphoric, and oxalic acid solutions are also damaging^7^. Metallic titanium with a density of 4.51 g/cm^3^ and mechanical properties comparable to mild steel, titanium has a very high strength to weight ratio: for this reason. Thus, it has become a backbone material in the aerospace industry^8^. Titanium is a very reactive metal: when unprotected in air, a thin TiO_2_ film (compact) is designed. It is usually adherent to the substrate and chemically stable in a variety of environments, and gives excellent corrosion resistance for titanium^8–10^ In general, all acidic solutions that are reducing in nature corrode titanium, unless they contain inhibitors.

Many industrial advances make use of acid solutions^11,12^ and so it is important to study it. Phosphoric acid is an essential industrial acid that is used to manufacture phosphatic fertilizers, acid pickling solutions, and in the food and beverage industry because of its outstanding chemical qualities. Metal corrosion can be prevented and hydrogen evolution reduced in corrosive conditions by using corrosion inhibitors^13–17^. The previous studies on the corrosion of titanium in environments containing phosphoric acid or phosphate were very limited.

Phosphoric acid was used as a depressant to separate the phosphate and carbonate minerals using the flotation process^18^. Flotation has a high economic feasibility for the beneficiation of valuable minerals when their principal ore bodies are magnesium (Mg) carbonates like dolomite and magnesite^19^. Magnesite (MgCO_3_), a common magnesium-bearing carbonate mineral, is one of the key materials of industrial magnesium smelting and source in the world for magnesia refractory production.

Recent research on the interactions of phosphoric acid with sparingly semi-soluble minerals (carbonate, phosphate) carried out by Filippov and co-workers showed the formation of a phosphate passivation film on the surface of phosphate minerals (apatite) which alters corrosion of the mineral by phosphoric acid^18,20,21^. Extending this approach to the corrosion inhibition of alloys would be an attractive and beneficial approach given the easy and cheap availability of mineral materials such as carbonates.

To our knowledge, no corrosion studies have been done using magnesite mineral as an inhibitor for Ti-alloy in phosphoric acid solution. The aim of this work is to evaluate the performance of magnesite material as a natural environmental inhibitor for Ti-6Al-4V alloy in phosphoric acid with different concentrations (0.001–0.5 M). In addition, the techniques used for the corrosion study may help to understand the phosphoric acid interaction mechanism with the mineral surface to refine the use of these reagents for mineral flotation.

## Materials and methods

The Ti-6Al-4V alloy, which has a cross-sectional area of 0.196 cm^2^ was purchased from Johnson and Matthey (England) with the chemical compositions listed in Table 1A. Magnesite sample was obtained from Egypt's Cairo-Suez Road. After that, the rock was crushed and finely ground with hammer mill first and then grinded more in a mortar before being used. Table 1B shows the XRF composition of magnesite. The sample presents a high purity mineral with the presence of a low amount of silica and probably iron oxides.

**Table 1:** Chemical composition of (A) Ti-6Al-4V (wt%) and (B) of magnesite.

Al	V	Fe	C	O	N	Ti				
(A)										
5.7	3.85	0.18	0.038	0.106	0.035	Balance				

Mg	Si	P	Cl	Ca	Ti	Mn	Fe	Zn	Nb	Mo
(B)										
40.1 9	2.23	0.23	0.08	2.98	0.04	0.78	9.59	0.03	0.01	0.01

The electrode (Ti alloy) was polished with SiC sheets (600–2400 grit), then washed with triple-distilled water, cleaned in an ultrasonic bath with acetone, and dried in the air. The working electrode (WE) is a Ti-6Al-4V alloy, the counter electrode (CE) is a platinum sheet, and the reference electrode (RE) is Ag/AgCl. The working electrode (Ti alloy) is immersed in a 100 mL solution of water and various phosphoric acid concentrations (0.001–0.5 M) with and without 3 g of magnesite mineral for 1 h with agitation at 245 rpm which means 3% magnesite as an inhibitor.

The test solution was prepared from different concentrations of phosphoric acid ranging from 0.001 to 0.5 M (Sigma Aldrich) with or without a 3% magnesite inhibitor (Sigma Aldrich). Triple distilled water was used to prepare all of the solutions.

The EC-Lab SP 150 Potentiostat electrochemical device was utilized to perform the electrochemical measurements. At a scan rate of 1 mV/s, potentiodynamic polarization tests were performed in the range from -1 to 1.5 V. The open-circuit potentials (OCP) were stabilized for 60 min before beginning the potentiodynamic experiments. Electrochemical impedance spectroscopy (EIS) was done in the frequency range of 100 mHz–100 kHz with a perturbation amplitude of 5 mV. All of the tests were repeated two or three times to ensure that the results were consistent. The scanning electron microscope (SEM) used was a Quanta 250 FEG (Field Emission Gun) with an Energy Dispersive X-ray spectroscopy (EDX) unit (FEI company, Netherlands).

Shimadzu Corporation's IR-Affinity-1, serial A21374701135S1, was utilized to measure FTIR. Cu-radiation (= 1.542) at 45 K.V., 35 M.A., and scanning speed 0.04°/s were employed on a PAN analytical X-Ray Diffraction apparatus type X-Pert PRO with secondary Monochromator.

The major and trace elements were determined using a Philips X-ray fluorescence equipment, model PW/2404, with Rh target and six analyzing crystals. Ca, Fe, K, Ti, Mn, and other trace elements from Nickel to Uranium were estimated using crystals (LIF-200), (LIF-220), whereas Mg and Na were determined using crystals (TIAP). Crystal (Ge) was utilized to estimate P, crystal (PET) was used to determine Si and Al, and PXI was used to determine Na and Mg. The concentrations of the components under investigation were determined using software Kernl X-44.

The pH of the phosphoric acid solution is measured by a pH meter (Hanna) after calibration with standard solutions of pH 4.01, 7.00 and 10.0.

Raman spectra were collected at wavenumber in the range of 200–4000 cm^−1^ by RXN4 spectrometer (Kaiser) at a laser excitation wave-length of 532 nm, with a fiber-optic Raman sampling probe. The sapphire-head probe gives Raman peaks at 377, 417, 447, 576 and 749 cm^−1^.

The Raman spectroscopy experiment is done for 3 g of the sample of magnesite in 100 ml of deionized water or phosphoric acid solutions at concentrations of 0.001, 0.01, 0.1, 0.25 and 0.5 M, and stirred at 200–300 rpm during 15 min.

**Figure 1:**
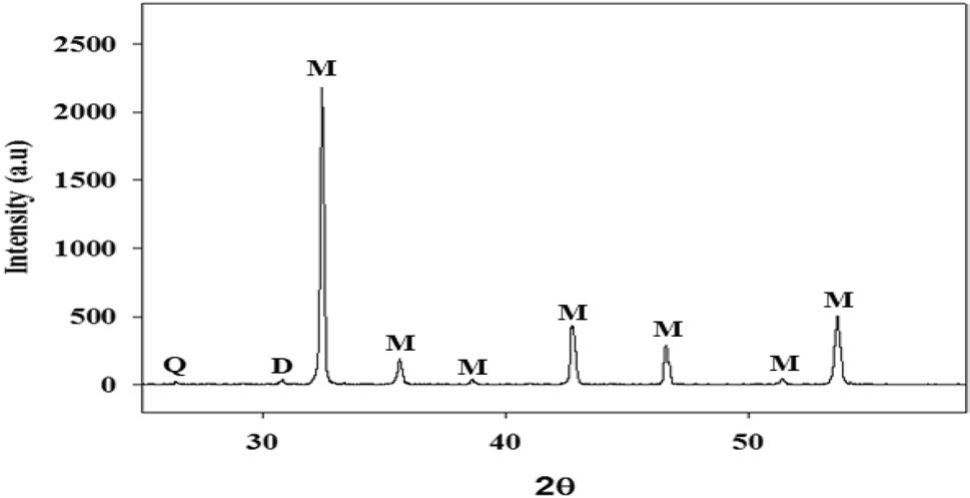
XRD analysis of magnesite sample: M = Magnesite, Q = Quartz, D = Dolomite.

The magnesite sample chemical and mineralogical compositions are determined to check its purity. The XRD (Fig. 1) analysis show that the sample is relatively pure and doesn’t contain other minerals in significant proportion except for a small amount of quartz and dolomite.

XRD was used to identify the sample's phase as it was prepared^22^. The typical XRD pattern is displayed in Fig. 1. Each peak can be correlated with the MgCO_3_ hexagonal phase. The report value (JCPDS Card Files, No 08-0479, a = 4.633 Å, c = 15.015 Å) is comparable with the lattice constants derived using the pattern (a = 4.63 Å, c = 14.93 Å). The strong peaks' strength indicates how intensely crystalline the magnesıte is. This pattern shows no further imperfections.

## Results and discussion

### Surfaces morphology and characterization

**Figure 2:**
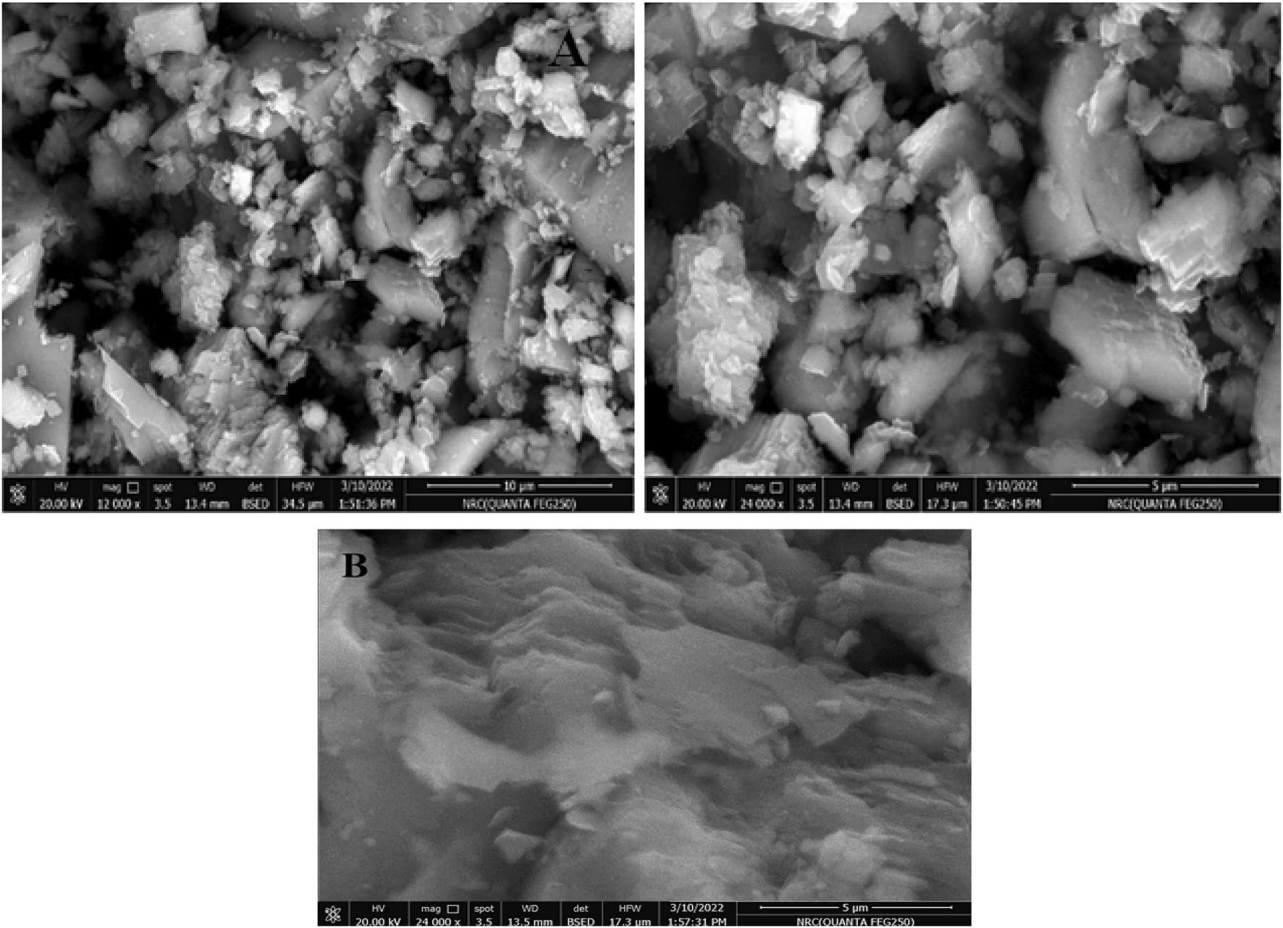
SEM images with different magnifications of magnesite sample (**A**) before and (**B**) after treatment in 0.5 M phosphoric acid solution after 1 h of immersion.

Figure 2A shows the SEM photos of magnesite rock mineral with different magnifications before immersion in phosphoric acid solution. It shows that the magnesite shape is like thick wood and flat. Figure 2B displays the SEM image as an example of a magnesite sample after treatment in 0.5 M phosphoric acid solution after 1 h of immersion and it shows compact film.

**Figure 3:**
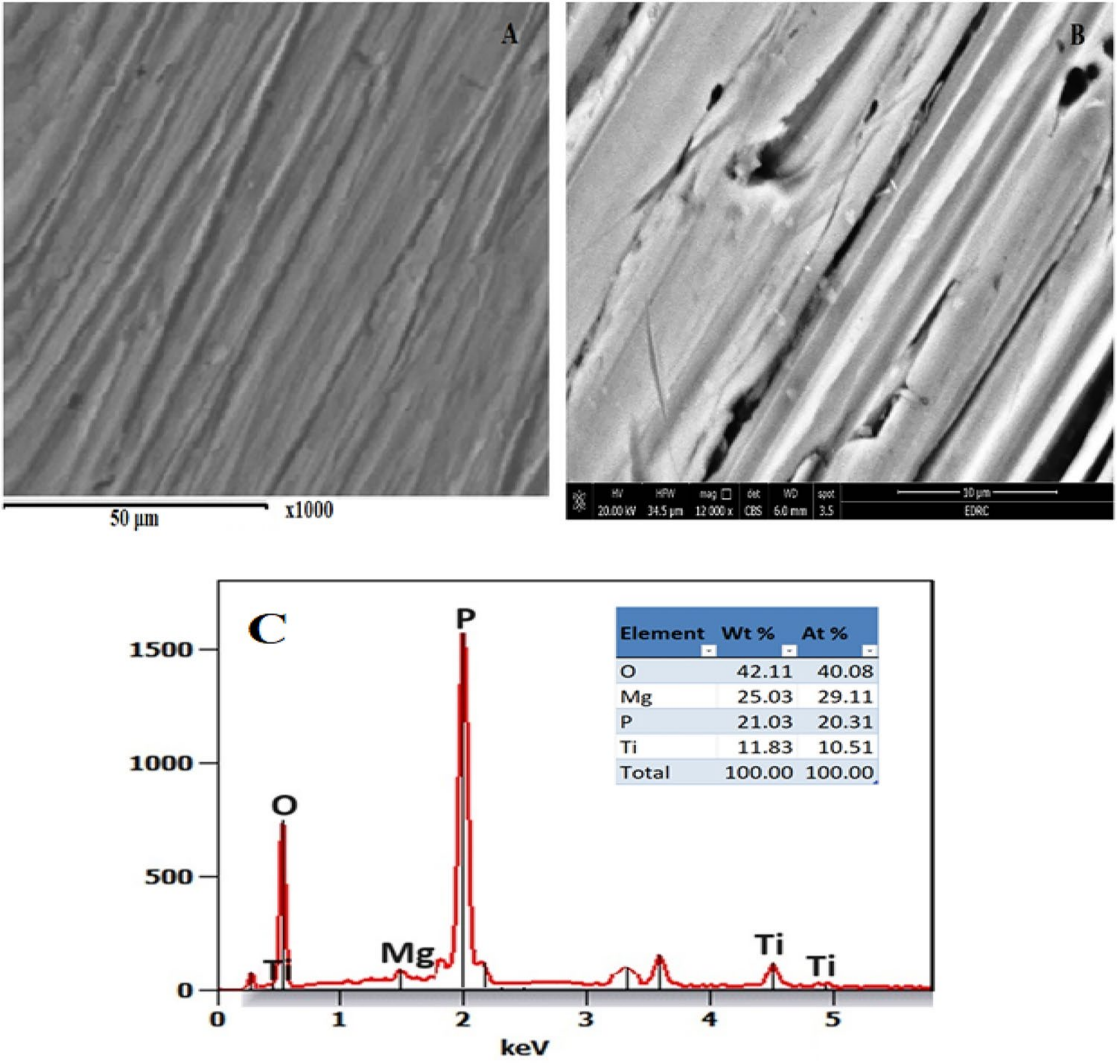
SEM images of (**A**) Ti alloy surface^23^; (**B**) after adding magnesite sample in 0.5 M phosphoric acid solution after 1 h of immersion with its (**C**) EDX analysis.

Figure 3A presents a pure Ti alloy surface which shows some scratches from polishing and Fig. 3B is for Ti alloy surface after adding magnesite sample in 0.5 M phosphoric acid solution after 1 h of immersion which shows a thick film of magnesium phosphate formed. Figure 3B presented the EDX analysis which confirmed the presence of magnesium phosphate film with titanium oxide on the alloy surface.

### Electrochemical impedance measurements

**Figure 4:**
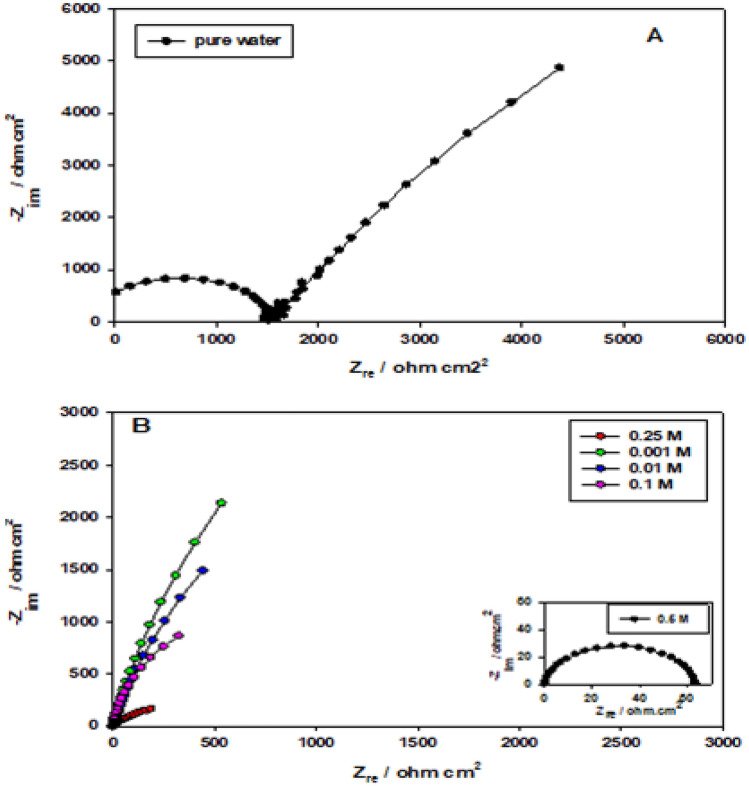
Nyquist plots of Ti-6Al-4V alloy (**A**) in water and (**B**) in phosphoric acid of different concentrations at 1 h of immersion without magnesite.

Nyquist plots (Fig. 4) of Ti-6Al-4V alloy in water or in phosphoric acid of different concentrations (0.001–0.5 M) after 1 h immersion time without magnesite were done to compare the tested alloy before and after adding magnesite. As you see in Fig. 4 the alloy in water only works with both adsorption (semicircle) and diffusion (line) mechanism, however, on phosphoric acid, it is only adsorption mechanism (semicircle)^10^.

**Figure 5:**
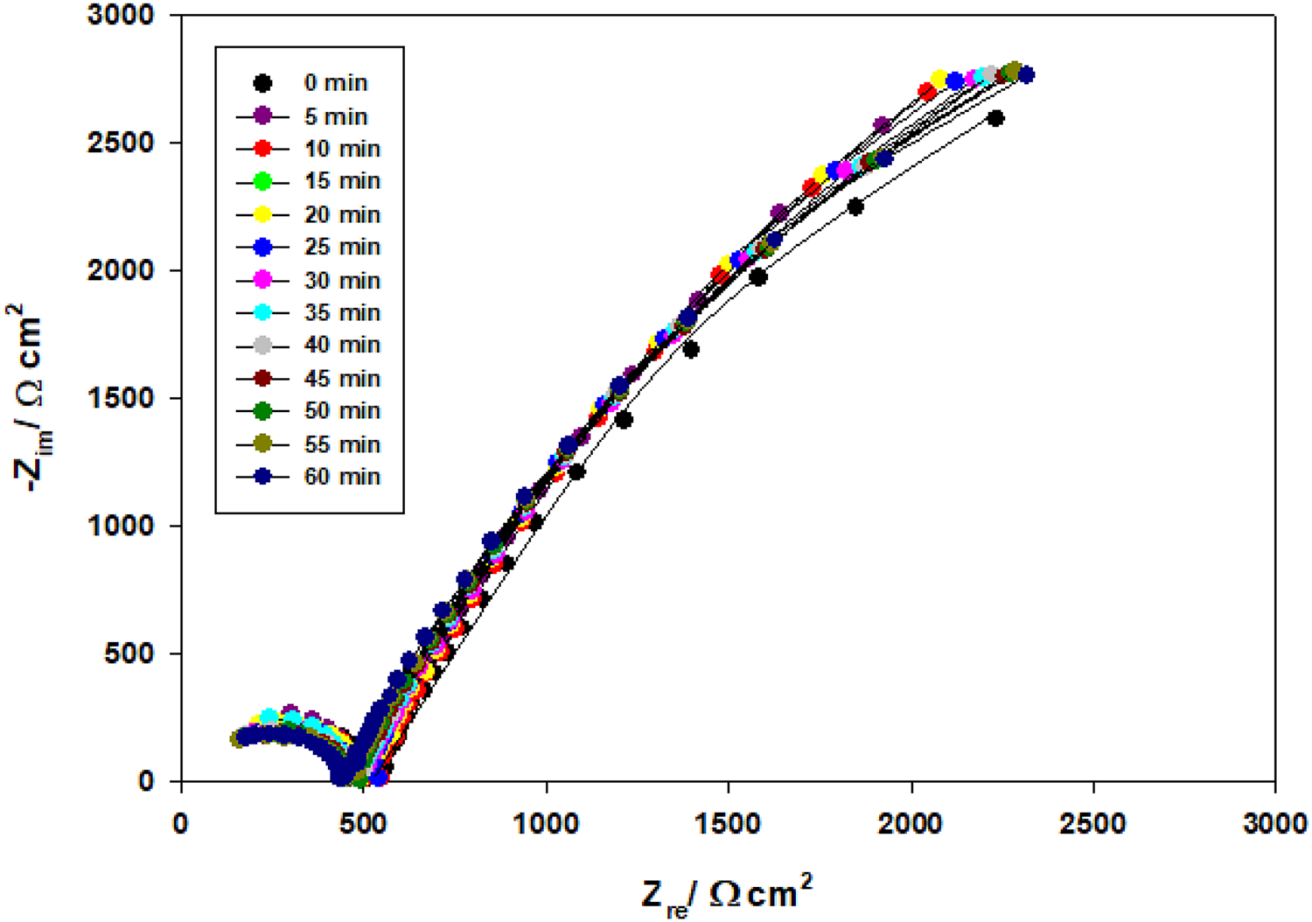
Nyquist plots of Ti-6Al-4V alloy in pure water containing 3% magnesite as a function of immersion time.

Electrochemical impedance spectroscopy (EIS) technique was performed in pure water containing 3% magnesite as a function of immersion time (0–60 min) and the results are presented in Fig. 5 (magnesite is sparingly soluble in water). Adding magnesite is in order to improve the corrosion resistance of the passive film formed on Ti-6Al-4V alloy. The impedance value of magnesite as an inhibitor for titanium alloy enlarged with immersion time due to magnesite dissolution and precipitation as a carbonate in water, as well as thickening of the passive TiO_2_ coating on the alloy surface^4,24–26^.

1 $${\text{MgCO}}_{{3}} = {\text{ Mg}}^{{{2} + }} + {\text{ CO}}_{{3}}^{{{2} - }}$$

Magnesite slightly dissolves with release of Mg^2+^ cations that cause precipitates to be developed on the metal's surface, forming a protective coating. Hard water with high calcium and magnesium content is less corrosive than soft water because the salts in hard water tend to precipitate on the metal's surface and form a protective coating^26^.

**Figure 6:**
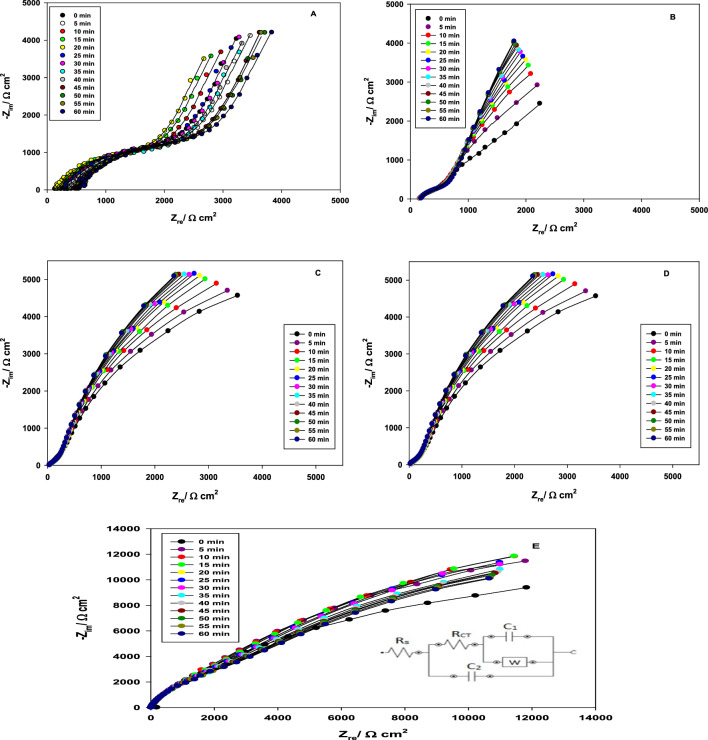
Nyquist plots of Ti-6Al-4V alloy in phosphoric acid of different concentrations (A:0.001;B:0.01;C:0.1;D:0.25;E:0.5 M) containing 3% magnesite as a function of immersion time and inset is the equivalent circuits used for modeling of experimental EIS data.

Electrochemical impedance spectroscopy technique is done to investigate the protective effect and the mechanism of reaction between magnesite mineral and phosphoric acid^10^. The impedance diagram for Ti-6Al-4V alloy in phosphoric acid with different concentrations (0.001–0.5 M) contains 3% magnesite as a function of immersion time represented in Fig. 6. The Nyquist plots show the same trend as compared to pure water. The Nyquist plots also reveal that magnesite mineral as an inhibitor for Ti-6Al-4V alloy has a high corrosion resistance at different (0.001–0.5 M) H_3_PO_4_ acid concentrations, indicating a thicker protective magnesium phosphate film is formed by adsorption on the alloy surface. The impedance value increases with further increasing H_3_PO_4_ concentration or increasing the immersion time reflecting the passivation of the oxide film on the Ti-6Al-4V alloy surface forming magnesium phosphate film with TiO_2_ film.

The reaction for the oxide film on Ti-6Al-4V alloy in phosphoric acid solution might be written as^4^:

2 $${\text{TiO}}_{{2}} + {\text{ H}}_{{2}} {\text{O }} + {\text{ H}}^{ + } = {\text{Ti }}\left( {{\text{OH}}} \right)^{{{3} + }}$$

The reaction between magnesite and dilute phosphoric acid at room temperature can be described by the equation.

3 $${\text{2 H}}_{{3}} {\text{PO}}_{{{4}({\text{aq}})}} + {\text{ 3 MgCO}}_{{{3}({\text{s}})}} = {\text{Mg}}_{{3}} \left( {{\text{PO}}_{{4}} } \right)_{{{2}\left( {\text{s}} \right)}} + {\text{ 3 CO}}_{{{2}({\text{g}})}} + {\text{ 3 H}}_{{2}} {\text{O}}_{{({\text{l}})}}$$

The extent of the reaction depends on H_3_PO_4_ concentration, pH, relative amounts of solid and liquid phases, temperature, and reaction time^27^. The best fit model used to obtain the parameters in Tables 2 and 3, is the Randles circuit with two time constant as represented in Fig. 6 for without and with magnesite mineral as an inhibitor for the Ti-6Al-4V alloy using EC program software with an average error of 4%. It comprises of a parallel combination of a capacitors (C_1_, C_2_) as a constant phase element (CPE) that is parallel to the surface film's polarization resistance (R_CT_), Warburg impedance (W) due to diffusion mechanism and both of them are connected to the solution resistance (R_s_). Because of the non-homogeneity of the electrode surface, CPE^11,29–35^ is utilized instead of capacitances in the two circuits [C_1_ (corresponding to inner layer) and C_2_ (corresponding to outer layer)].

4 $${\text{Z}}_{{{\text{CPE}}}} = \, \left[ {{\text{C }}\left( {{\text{j}}\omega } \right)^{\alpha } } \right]^{{ - {1}}}$$

where α is an exponent account for surface heterogeneity, 0 ≤ α ≤ 1, j is the imaginary number (j = (− 1)^1/2^), ω = 2πf is the angular frequency in rad/s, f is the frequency in Hz = S^−1^.

**Table 2:** Electrochemical impedance parameters of Ti-6Al-4V alloy in pure water and phosphoric acid of different concentrations (0.001–0.5 M) after immersion for 1 h.

Conc M	R_s_ Ω cm^2^	R_CT_ Ω cm^2^	C_1_ μF cm^−2^	W Ω cm^2^ s^−1/2^	C_2_ μF cm^−2^
0.000	2632	4811	2.30	19.5	3.2
0.001	2102	3562	10.5	17.8	3.7
0.010	362	2810	11.9	15.3	4.2
0.100	38.2	1412	13.5	12.5	6.8
0.250	24.1	532.0	19.0	9.30	8.1
0.500	15.1	63.70	23.0	6.50	8.2

**Table 3:** Electrochemical impedance parameters of Ti-6Al-4V alloy in pure water and phosphoric acid solution of different concentrations (0.001–0.5 M) after immersion for 1 h containing 3% magnesite.

Conc	Time min	R_s_ Ω cm^2^	R_CT_ Ω cm^2^	C_1_ µF cm^−2^	W × 10^–5^ Ω cm^2^ s^−1/2^	C_2_ µF cm^−2^	IE%
0.00	0	1530.9	4770	340.34	45.2	160.8	–
	5	2010.5	4827	338.69	45.2	158.4	–
	10	2607.2	4875	338.14	51.1	154.7	–
	15	2618.7	4924	339.02	49.9	146.3	–
	20	2682.7	4928	337.81	46.8	145.2	–
	25	2719.5	4934	336.82	40.3	143.2	–
	30	2725.9	4941	337.26	46.6	141.3	–
	40	2852.6	4984	336.82	47.3	131.5	–
	50	2939.9	5047	338.03	42.7	132.4	–
	60	3102.7	5070	337.48	45.8	132.1	5.11
0.001	0	791.92	5920	309.4	30.4	185.1	–
	5	789.34	6024	307.9	28.6	184.1	–
	10	791.92	6124	307.4	28.7	183.8	–
	15	823.26	6338	308.2	28.9	183.1	–
	20	819.97	6504	307.1	28.2	182.9	–
	25	881.02	6742	306.2	28.2	182.4	–
	30	1258.6	6924	306.6	27.8	181.8	–
	40	1615.3	7128	306.2	27.1	181.4	–
	50	1782.6	7304	307.3	26.7	181.2	–
	60	2098.5	7520	306.8	26.3	180.4	52.60
0.01	0	149.85	6746	280.8	24.3	222.6	–
	5	156.83	6910	279.5	22.9	224.2	–
	10	160.26	7131	279.1	23.6	221.1	–
	15	165.85	7254	279.6	23.2	223.4	–
	20	170.07	7322	278.9	22.6	219.4	–
	25	173.54	7400	278.6	22.6	219.8	–
	30	182.25	7530	278.1	22.3	218.2	–
	40	185.24	7600	277.8	21.9	218.3	–
	50	186.71	7791	278.9	21.7	217.3	–
	60	198.18	7923	277.9	21.5	216.6	64.53
0.1	0	67	7013	218.8	14.9	244.8	–
	5	71	7264	215.1	14.7	246.6	–
	10	74	7611	214.8	14.8	243.2	–
	15	85	7782	214.5	14.8	245.7	–
	20	88	7890	214.5	14.8	241.3	–
	25	94	7986	214.4	15.5	241.7	–
	30	101	8121	214.6	15.3	240.0	–
	40	105	8411	214.5	15.4	240.1	–
	50	113	8719	214.4	15.5	239.0	–
	60	124	8812	214.4	16.2	238.2	83.98
0.25	0	53.24	11,828	14.8	9.09	381.0	–
	5	55.41	12,022	13.7	8.90	367.0	–
	10	62.12	12,551	11.8	8.72	356.0	–
	15	67.45	12,670	12.5	8.64	357.0	–
	20	70.23	12,756	10.8	8.52	361.0	–
	25	74.52	12,851	10.8	8.72	366.0	–
	30	79.54	12,966	10.6	8.29	356.0	–
	40	84.42	13,180	10.7	8.23	381.0	–
	50	91.12	13,360	10.5	8.27	383.0	–
	60	97.32	13,590	10.2	8.28	382.0	96.09
0.5	0	39.5	12,268	11.8	8.29	475.8	–
	5	44.01	12,438	10.9	8.09	458.2	–
	10	51.7	12,629	9.40	7.93	445.1	–
	15	53.3	12,725	10.0	7.85	446.2	–
	20	56.8	13,030	9.67	7.75	451.2	–
	25	59.1	13,456	8.62	7.93	457.2	–
	30	64.6	14,452	8.45	7.54	445.2	–
	40	67.4	15,187	8.24	7.48	478.2	–
	50	72.2	16,191	8.58	7.52	475.1	–
	60	77.85	17,693	8.41	7.53	411.5	99.64

The results of EIS fitting analysis are given in Tables 2 and 3. It is clear as given in Table 2, that the impedance has lower values without adding magnesite mineral which acts as an effective inhibitor in aggressive acid medium and leads to a decrease in hydrogen evolution as seen in Table 3. It can be seen clearly that the mechanism of the reaction changed totally from diffusion to adsorption, where in water, it works with both mechanisms’ diffusion and adsorption^10^. After increasing phosphoric acid concentration, the adsorption behavior enhanced and the diffusion process reduced sharply after adding the mineral. This is due to the increase in the formation of insoluble film of magnesium phosphate that increases the charge transfer resistance and decreases Warburg impedance sharply with increasing phosphoric acid concentration and increasing hydrogen evolution. Also, the values of the capacitances, C_1_ and C_2_ are inversely proportional to the resistance value which insures thickening as obtained by its decrease with the increase of the resistance value. The capacitance is related to the thickness, since the passive oxide film can be considered as a dielectric plate capacitor, the passive film thickness (d) in cm is related to the capacitance (C) by the Eq. (5)^34^:

5 $${\text{d }} = \, \varepsilon_{{\text{o}}} \varepsilon_{{\text{r}}} \left( {{\text{A}}/{\text{C}}} \right)$$

where ε_o_ is the vacuum permittivity (8.85 × 10^−12^ F cm^−1^), ε_r_ is the relative dielectric constant of the film and A is the electrode area in cm^2^. Although the actual value of ε_r_ within the film is difficult to estimate, a change of C can be used as an indicator for change in the film thickness. Hence, the reciprocal capacitance (1/C) of the surface film is directly proportional to its thickness.

Thus, the inhibition efficiency of magnesite is calculated by^11,29,30^:

5 $${\text{IE }}\% \, = { 1 }{-}{\text{ R}}_{{\text{p}}}^{ \circ } /{\text{ R}}_{{\text{p}}} \times { 1}00$$

where Rº_p_ and R_p_ are the total resistances for the Ti-6Al-4V alloy without and with 3% magnesite inhibitor, respectively.

It was found that the inhibition efficiency of magnesite after adding to water or phosphoric acid is 5.11% in water, 52.6% at 0.001 M, 64.53% at 0.01 M, 83.98% at 0.1 M, 96.09% at 0.25 M reaching to the highest inhibition efficiency of 99.64% at 0.5 M. This ensures the effectiveness of magnesite rock material especially at high concentration of phosphoric acid with high hydrogen evolution.

### Potentiodynamic polarization measurements

**Figure 7:**
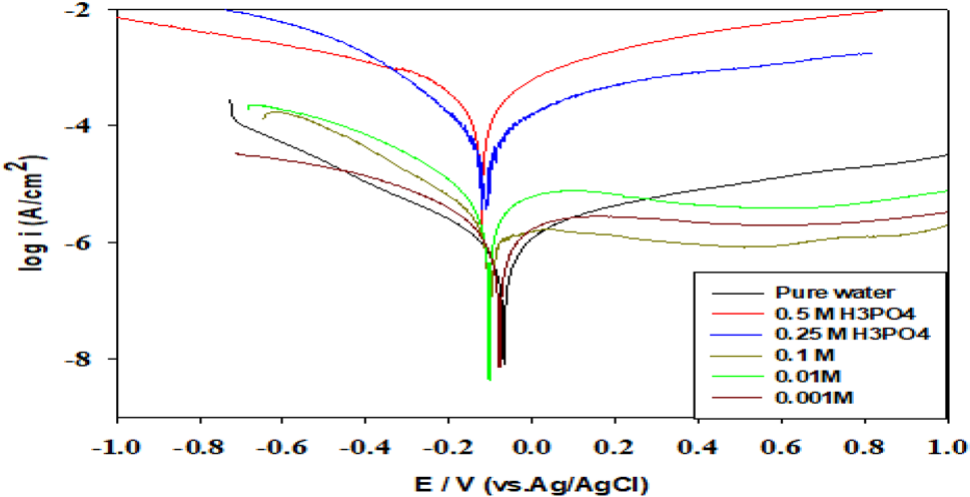
Polarization curves of Ti-6Al-4V alloy in phosphoric acid of different concentrations (0.001–0.5 M).

**Figure 8:**
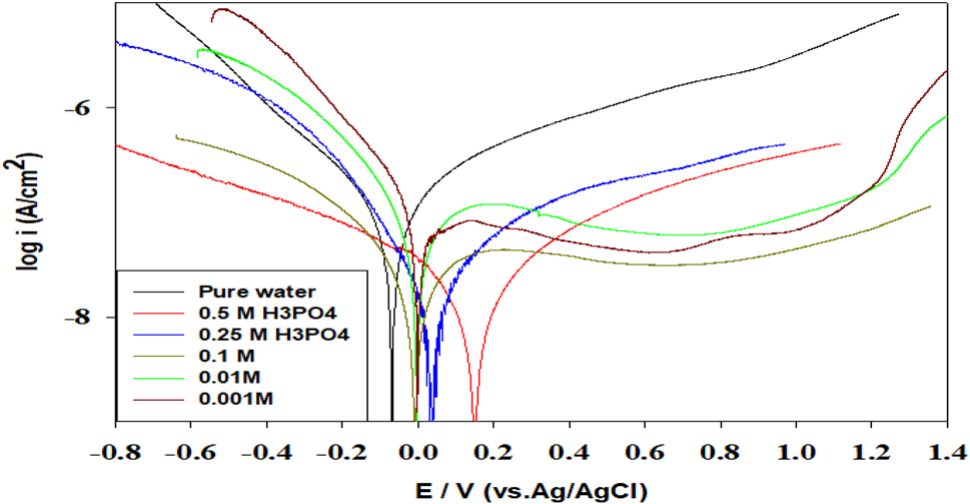
Polarization curves of Ti-6Al-4V alloy in phosphoric acid of different concentrations (0.001–0.5 M) containing 3% magnesite.

To confirm further the EIS results, potentiodynamic polarization utilized for the Ti-6Al-4V alloy in H_3_PO_4_ acid solution of different concentrations (0.001–0.5 M) without and with 3% magnesite as represented in Figs. 7, 8, respectively.

In the absence of magnesite, the concentration of phosphoric acid causes the corrosion potential (E_corr_) to shift towards a more negative potential, reflecting the inhibition of the cathodic reaction, which is the hydrogen evolution as represented in Fig. 7. However, higher acid concentrations cause the alloy to dissolve, raising the corrosion current density. It is clear from Fig. 8 that the addition of 3% magnesite decreased the current density value, shifting the corrosion potential towards a positive direction, reaching a value of 0.012 μA/cm^2^ and 149 mV, respectively. Magnesite inhibitor lowers corrosion, oxygen and hydrogen evolution by decreasing the anodic and cathodic branches (mixed inhibitor) protecting the surface due to good adsorption of magnesite mineral on Ti-6Al-4V alloy in H_3_PO_4_ acid, which prevents titanium oxide film dissolution in phosphoric acid.

The corrosion inhibition efficiency IE %^31,32^ of the Ti-6Al-4V alloy in phosphoric acid of different concentrations (0.001–0.5 M) containing 3% magnesite were calculated from corrosion current densities values in Table 4 using the following Eq. (6).

6 $${\text{IE }}\% \, = { 1 }{-}{\text{ i}}_{{{\text{corr}}}} /{\text{ i}}_{{{\text{corr}}^{ \circ } }} \times { 1}00$$

where i_corrº_ and i_corr_ are the corrosion current densities for the Ti-6Al-4V alloy without and with 3% magnesite inhibitor, respectively^28,29^. The corrosion parameters derived from the polarization curves such as, corrosion potential (E_corr_), corrosion current density (I_corr_), and inhibition efficiency IE% were recorded in Table (4).

**Table 4:** Electrochemical parameters and inhibition efficiency obtained by polarization tests.

	Conc/M	βa	βc	E_corr_/mV	I_corr_/μA/cm^2^	IE%
Without magnesite	0.00	387.7	213.5	− 71	1.25	–
	0.001	343.2	210.1	− 80	1.10	–
	0.01	299.1	180.5	− 104	6.87	–
	0.10	278.5	193.5	− 101	2.88	–
	0.25	362.4	205.3	− 108	134	–
	0.5	351.2	211.6	− 117	219	–
With 3 % magnesite	0.00	80.1	82.2	− 93.7	0.087	93.04
	0.001	88.3	79.5	− 22.8	0.076	93.09
	0.01	78.2	70.2	− 15.2	0.075	98.91
	0.10	66.8	79.3	− 7.12	0.031	98.92
	0.25	64.3	56.2	34.2	0.015	99.99
	0.50	66.9	58.6	149	0.012	99.99

It is clear that the efficiency is so high reaching 99.99% at high concentrations of 0.25 and 0.5 M of phosphoric acid.

### Raman kinetic analysis for magnesite interaction with phosphoric acid

The dissolution procedure of magnesite and other carbonate minerals in phosphoric acid solution is governed by its acidity^20,21,33,36–38^.

The kinetics of interaction of the salt type carbonate and phosphate minerals (calcite and apatite) with the phosphoric acid solutions were investigated in details using in situ and ex-situ Raman spectroscopy^16,17,37,38^ without the Ti alloy. The studies performed allowed to deduce the formation of dicalcium phosphate on the surface of apatite while the surface of calcite was continuously renewed allowing a differential behavior in flotation due to the surface passivation of apatite in a narrow interval of contact time and acid concentration^18,37^. Although the previous work revealed the neoformation on the mineral surface they do not provide the mechanism of such behavior.

**Figure 9:**
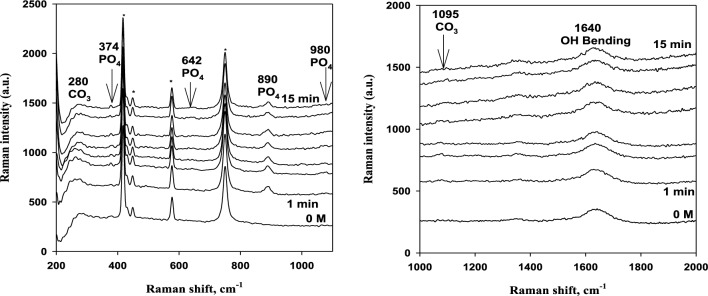
Raman spectra in the region from 200 to 2000 cm^−1^ for 3% Magnesite in contact with 0.1 M phosphoric acid solution for 15 min immersion (* probe peaks).

**Figure 10:**
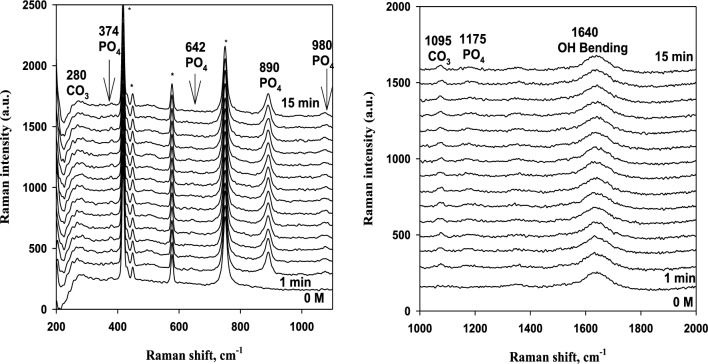
Raman spectra in the region from 200 to 2000 cm^−1^ for 3% Magnesite in contact with 0.25 M phosphoric acid solution for 15 min immersion (* probe peaks).

**Figure 11:**
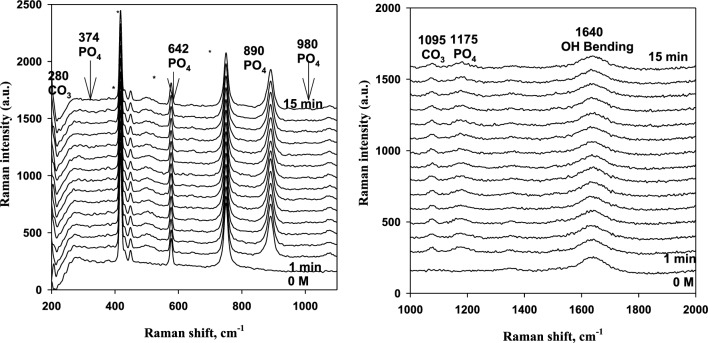
Raman spectra in the region from 200 to 2000 cm^−1^ for 3% Magnesite in contact with 0.5 M phosphoric acid solution for 15 min immersion (* probe peaks).

Herein, the Raman spectra of magnesite were measured using the same methodology^20,21^, i.e. in situ as a function of contact time with solutions of deionized water and phosphoric acid of different concentrations. The sharp peak at 890 cm^−1^ belongs to phosphoric acid at 0.1 to 0.5 M. The position and intensity of all peaks remains more or less constant with time. This can be seen in Figs. 9, 10 and 11 as an example. Generally, the region from 200 to 400 cm^−1^ is due to Mg–O modes or Mg–O vibrations, and at 890 cm^−1^ is due to PO_4_ vibrations. Also, the peaks at 980 cm^−1^ are so small and are due to phosphate vibrations. The peaks at 1095 cm^−1^ are due to carbonate vibrations and at 1640 cm^−1^ are due to OH bending.

For deionized water, 0.001 M, and 0.01 M phosphoric acid solution, 1095 and 280 cm^−1^ peaks are weak and they are due to carbonate vibrations. A new small peak appears at 980 cm^−1^ and 1175 cm^−1^ at only 0.25 M and 0.5 M, due to phosphate vibrations and formation. The carbonate dissolved in the aggressive acid medium and converted to phosphate giving its peaks due to the adsorption of phosphate film increases as stated before on EIS data.

This confirms EIS and polarization data, which showed that the reaction mechanism changed completely from diffusion of carbonates and phosphates to adsorption with increasing acid concentration due to phosphate film formation by adsorption at high concentrations and carbonate dissolution in this aggressive medium. Also, diffusion is limited and decreases sharply at the highest concentration. So, increasing phosphoric acid concentration enhanced the adsorption behavior and reduced the diffusion process. Thus, carbonate peaks disappear and phosphate peaks appear in Raman spectra.

**Figure 12:**
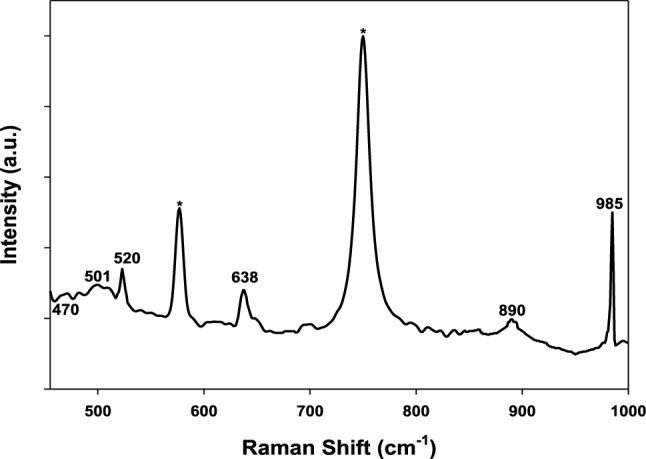
Raman spectra in the region from 470 to 1000 cm^−1^ for the surface film on the Ti alloy in 0.5 M phosphoric acid solution containing 3% Magnesite after immersion for 1 h (* probe peaks).

Figure 12 shows the Raman spectra in the region 450 to 1000 cm^−1^ for the film growing on the Ti alloy in 0.5 M phosphoric acid solution containing 3% magnesite after immersion for 1 h. It shows the peaks of TiO_2_ film at 520 and 638 cm^−1^^39^ and the peaks at 470 and 985 cm^−1^ are for magnesium phosphate film ensuring the formation of the film on the alloy surface.

So, at the highest concentration of phosphoric acid, the impedance value increases and corrosion current density decreases well due to phosphate film formation on alloy surface which is confirmed by the appearance of phosphate peak in Raman spectra at this concentration.

### FTIR analysis for phosphoric acid without and with 3% magnesite

The FTIR measurement was taken for phosphoric acid only without and with 3% magnesite in solution to confirm the formation of the magnesium phosphate film.

**Figure 13:**
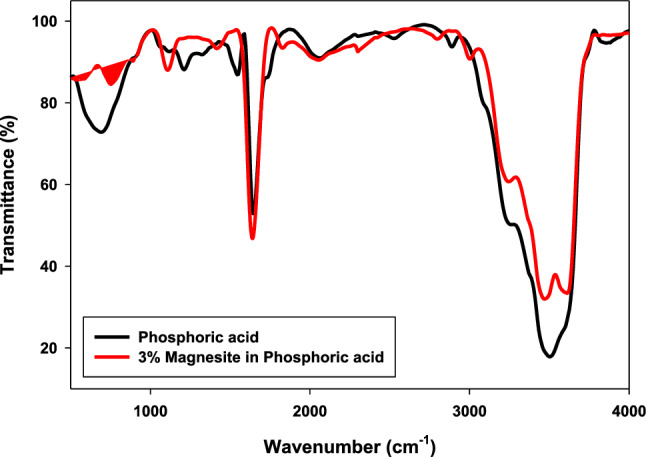
FTIR for phosphoric acid solution without and with 3% magnesite.

The Fourier transform infrared spectra (FTIR) are shown in Fig. 13, and were recorded to understand the chemical and molecular interactions between 0.5 M phosphoric acid and 3% magnesite. It is clear that after adding magnesite, there are new bands at 683 cm^−1^ due to magnesium phosphate (Newberyite) formation^36^. Other bands at 1108 and 1419 cm^−1^ are due to carbonates. Also, all bands higher than 1600 cm^−1^ are due to H–O–H bending or stretching. This confirms magnesium phosphate film formation.

Finally, all results obtained using all techniques and analyses ensured the excellent protective behavior of magnesite inhibitor for Ti-6Al-4V alloy in phosphoric acid of different concentrations (0.001–0.5 M) and considered as a novel natural, low cost and widely available material used in industrial applications.

Moreover, the obtained data allow us to deduce the diffusion limited mechanism of the magnesite interaction with phosphoric acid at low concentration and the enhanced adsorption behavior when the phosphoric acid concentration increases. The formation of magnesium phosphate passivation film on the magnesite surface controls the Mg^2+^ concentration in the solution for a better inhibition action on the Ti-alloy surface. Magnesite mineral dissolves with increasing acid concentration and converted to magnesium phosphate which is a good film that works on the alloy surface protection by adsorption behavior and diffusion limited behavior as confirmed by all done techniques in this work. This suggests that magnesite natural mineral can be used as an effective inhibitor even at high acid concentrations.

## Conclusions

Based on the results of potentiodynamic polarization and EIS measurements, the following conclusions are drawn in this study:

1. The electrochemical corrosion resistance of the Ti-6Al-4V alloy in phosphoric acid increases with immersion time.
2. The corrosion resistance of Ti-6Al-4V alloy in phosphoric acid shows that magnesite mineral behaves as an inhibitor, and that the corrosion resistance increases with increasing phosphoric acid concentration containing it and the hydrogen evolution decreases. This means that magnesite mineral protects the alloy surface from the corrosive acid medium.
3. The excellent inhibition effect of magnesite was attributed to the formed protective film, consisting of the insoluble magnesium phosphates.
4. The inhibition efficiency of the magnesite improved with the increase in the phosphoric acid concentrations.
